# Determinants of breast cancer in Ethiopia: a systematic review and meta-analysis

**DOI:** 10.3332/ecancer.2023.1624

**Published:** 2023-11-10

**Authors:** Lencho Kajela Solbana, Eshetu Ejeta Chaka

**Affiliations:** 1 College of Health Science, Assosa University, PO Box 018, Assosa, Ethiopia; 2 College of Medicine and Health Sciences, Ambo University, PO Box 19, Ambo, Ethiopia

**Keywords:** breast cancer, determinant, Ethiopia, women, meta-analysis

## Abstract

**Background:**

Breast cancer (BC) is the first leading cancer sharing about 25% of the cancer burden among women globally. This study aimed to identify the determinants of BC in Ethiopia.

**Methods:**

We comprehensively searched primary studies conducted in Ethiopia on associated factors of BC in PubMed, Cochrane Library, Hinari, Google, and Google Scholar) and available online until 2 June 2023. The necessary data were extracted from relevant studies and exported to STATA version 15 for analysis. The pooled odds ratio with its 95% confidence interval (CI) was estimated using a random effect model. The finding was reported following preferred reporting items for systematic reviews and meta-analyses guidelines.

**Results:**

Five studies with 1,819 participants (792 cases and 1,027 controls) were included. The significant determinants of BC were age at menarche <12 years (adjusted odds ratio (AOR) = 3.36, 95% CI: 1.68–5.04), post-menopause (AOR = 2.37, 95% CI: 1.67–3.06), ever breastfeeding (AOR = 0.28, 95% CI: 0.15–0.42), and family history of cancer (AOR = 2.39, 95% CI: 1.29–3.44).

**Conclusion:**

In Ethiopia, the significant determinants of BC among women were age at menarche <12 years, post-menopause, Ever breastfeeding, and family history of cancer. We recommend that the concerned organizations consider the aforementioned factors in addressing the problem of BC in Ethiopia by increasing community awareness, promoting breast self-examination, and developing programs to reduce the increasing burden of BC in the study setting.

## Introduction

Breast cancer (BC) is a group of diseases in which cells in breast tissue change and divide uncontrolled, typically resulting in a lump or mass [1, 2]. It can be invasive or non-invasive. The invasive BC is where the cancer cells are only found in the ducts, which is known as ductal carcinoma *in situ* (DCIS) and the non-invasive BC is where the cancer cells have grown into surrounding tissue [3]. Types of BC depend on which cells in the breast turn into cancer [2]. Most BCs begin in the lobules (milk glands) or in the ducts that connect the lobules to the nipple [1]. It can spread outside the breast through blood vessels and lymph vessels. When this happens, it is said to have metastasized [2]. Less frequently occurring types of BC also occur in breast tissues, these cancers are called sarcomas and lymphomas [3].

Globally, BC accounts for 12.5% of cancer in the general population and shares 25.8% of cancer cases among women in 2020 [4]. It is the fifth leading cause of cancer mortality worldwide [5]. The International Agency for Research on Cancer (IARC) estimates there were more than 2.26 million new cases of BC and 685,000 deaths from it worldwide in 2020 [6]. In Sub-Saharan African (SSA) countries, BC was the most common cancer type among women [7]. In SSA lack of human resources and service delivery such as difficulty accessing health care, diagnostic errors, poor management, and treatment costs were the principal health system factors that influenced the diagnosis and treatment of women with BC [8].

Low-income countries have poorly developed health systems including cancer services indeed need up-grading [9]. In 2014 World Health Organization (WHO) report indicated that cancer is an increasing public health burden for Ethiopia [10]. In 2018, BC was the first leading cancer type which accounts for 22.6% and 17.0% of the morbidity and mortality of the cancer burden in the country [11]. The finding of the trend analysis done from 2010 to 2019 indicates the common killer cancers in Ethiopia were leukemia, BC, cervical cancer, and stomach cancer [12]. The other trend analysis done from 2013 to 2019 at Hawassa University Comprehensive Specialized Hospital shows there was a continuous increment in BC cases at the hospital [13].

According to a previous qualitative study done in southwest Ethiopia, misdiagnosis of BC, long distance to referral facilities, high cost of diagnostic services, long waiting time for diagnostic tests, and lack of screening and diagnostic tests in local facilities were identified as health-system-related barriers for late diagnosis of BC [14]. On the other hand, according to a systematic review and meta-analysis done in 2020, only 36.72% of women practice breast self-examination [15]. According to a study done at Tikur Anbessa Specialized Hospital in 2015, the treatment outcome of BC was mainly poor, so health education and sensitization on prevention were suggested [16]. The finding of a study done in the same area in 2016 indicates were BC, uterine cancer, colorectal cancer, uterine cancer, cervical cancer, esophageal cancer, osteosarcoma, squamous cell carcinoma were the leading cancer types among women aged 25–49 years [17].

The prevalence of BC is found to be increasing in Ethiopia as some studies indicate [12, 13]. However, women's awareness about the disease was low and there was also a problem of late diagnosis of the disease [14]. Late diagnosis of BC leads to poor treatment outcomes [16]. The findings of previous studies done on determinants of BC were inconsistent; and may vary from study to study. Therefore, this study aimed to identify common determinants of BC among Ethiopian women.

## Methods

This study was conducted following preferred reporting items for systematic reviews and meta-analyses (PRISMA) guidelines.

### PECOS framework

The population, exposure, comparisons, outcome, study design (PECOS) framework of the study is described in Table 1 below.

### Protocol registration

The protocol for this study was registered to the International Prospective Registry of Systematic Reviews (PROSPERO) with registration number CRD42023431578.

### Search strategy and study selection

A comprehensive search of both published and unpublished primary studies was conducted using several databases (PubMed, Google Scholar, university research repository websites, Cochrane Library, Hinari, and Google). The search was done by combining various search phrases ((((((“Risk factor”[Title/Abstract]) OR (Determinant [Title/Abstract])) OR (“Associated factor”[Title/Abstract])) OR (“Modifiable factor”[Title/Abstract])) OR (Predictor [Title/Abstract])) AND ((“Breast cancer”[Title/Abstract]) OR (“Breast Neoplasms”[Mesh]))) AND (Ethiopia [Title/Abstract]).

### Eligibility criteria

**Figure 1.**
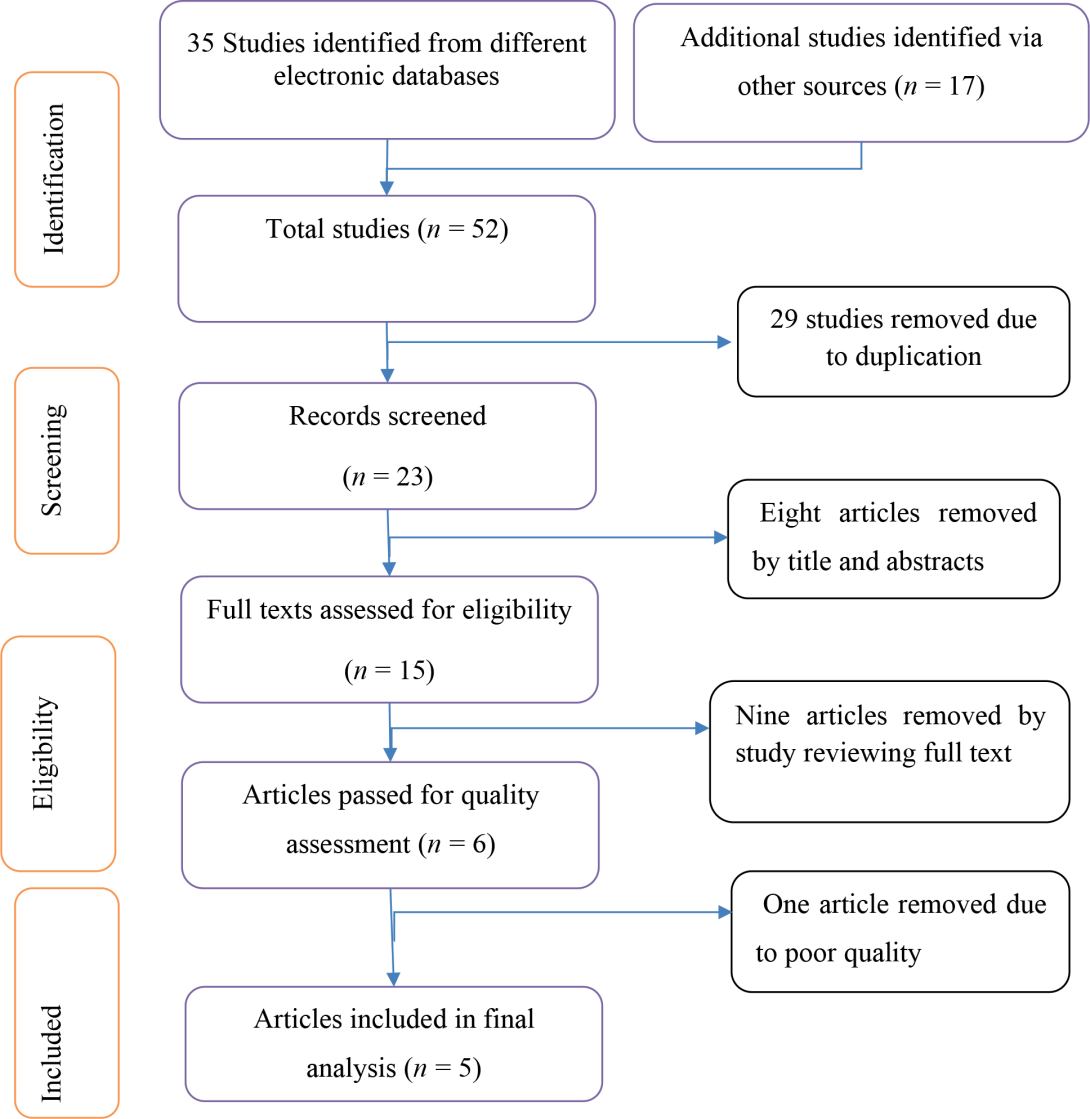
PRISMA flow diagram of the included studies.

Primary observational studies done on associated factors of BC conducted at any time on women in Ethiopia and published in the English language and available online until 2 June 2023, were identified. The identified articles were first evaluated for their titles and abstracts. Then relevant studies were further evaluated by reading their full text. The quality of individual studies was assessed using the Newcastle-Ottawa scale (NOS) for case-control study [18], articles having a scale ≥7 were included in this meta-analysis (Table 2 and Figure 1).

### Exposures of interest

- History of ever breastfeeding (Yes versus No)
- Family history of cancer (Yes versus No)
- Age at menarche (<12 versus >15 years)
- Menopause status (post-menopause versus pre-menopause)
- Body mass index (≥25 versus <18.5 kg/m^2^)

### Data extraction

Our review team prepared the data extraction form in Microsoft Excel before data extraction. Next, the necessary data from each included paper was extracted by both authors independently. The components of the data extraction formats include; the name of the first author, publication year, year of study, study design, study setting, study area, sample size, name of health facility (when institution-based), associated factors (or determinants), comparison group (reference group), adjusted odds ratio (AOR) with its lower level and upper level 95% confidence interval (CI) for the associated factors. In addition, when necessary data were not published together with the original studies, the corresponding author was requested to provide it.

### Outcome

This study has one primary outcome; which was determinants of BC.

### Statistical analysis and presenting findings

**Figure 2.**
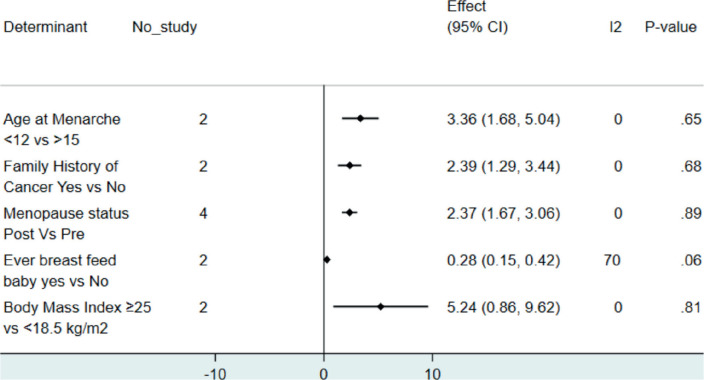
Forest plot revealing determinants of BC in Ethiopia.

The extracted data were exported to STATA version 15.0 software for analysis. The pooled odds ratio was conducted using a random effect model. When the 95% CI of the pooled odds ratio did not include 1, the factors were considered to be significantly associated (Figure 2). The characteristics of individual studies and the syntheses were presented using tables, figures, and statements.

## Results

### Study selection

A total of 52 studies (35 from electronic databases and 17 other sources) were identified. After 29 duplicates were removed, 23 studies were screened for titles and abstracts. Eight studies were excluded since found to be irrelevant and 15 studies were assessed for full text. Nine studies were excluded after reviewing full texts, and six studies were evaluated against NOS quality. Finally, one study was excluded by quality score and five studies were included in the final analysis (Table 2).

### Characteristics of the included studies

All the included studies were conducted from 2013 to 2020 [19–23]. The study design of all the studies was case-control, whose study area was Addis Ababa [19–23]. In addition, all studies were institution based [19–23]. The sample size of the studies ranges from 220 to 434, with varying control-to-case ratios among the included studies [19–23]. All the included studies were those identified from different journals (published studies) (Table 3).

### Synthesis of results

In the present study, five studies having 1,819 total participants (792 cases and 1,027 controls) were included. The significant determinants of BC were age at menarche <12 years (AOR = 3.36, 95% CI: 1.68–5.04), post-menopause (AOR = 2.37, 95% CI: 1.67–3.06), ever breastfeeding (AOR = 0.28, 95% CI: 0.15–0.42), and family history of cancer (AOR = 2.39, 95% CI: 1.29–3.44) (Figure 2).

## Discussion

This study aimed to identify determinants of BC in Ethiopian women. Age at menarche <12 years, post menopause, not ever breastfeeding a baby, and having a family history of cancer increased the odds of developing BC.

### Ever breastfeeding

It was identified that a history of ever breastfeeding decreased the odds of developing BC by 72% (AOR = 0.28, 95% CI: 0.15–0.42) which is supported by findings of previous meta-analyses done in other countries [25, 26]. Breastfeeding reduces BC risk biologically through lower levels of systemic estrogen and progesterone levels during breastfeeding and the excretion of estrogen and carcinogens from the breast ducts [21]. However, when breastfeeding stops, fatty tissue replaces the mammary gland, increasing the risk of BC [27].

For a variety of reasons, including working outside the home, employed Ethiopian women choose bottle feeding to breastfeeding [28]. Lack of workplace breastfeeding laws, arrangements, and support hinders mothers’ freedom to practice optimal breastfeeding, according to a 2021 study that compared breastfeeding in Ethiopia to the international standard [29]. Therefore, policymakers, the government, and other concerned bodies should pay close attention to the facilitation of arrangements to enable employed mothers to practice optimal breastfeeding upon return to work; strengthening breastfeeding has a dual benefit for mothers and their babies.

### Age at menarche <12 years old

The finding of this study indicates that age at menarche of <12 years old increased the odds of developing BC by 3.36 times more likely as compared to age at menarche after 15 years (AOR = 3.36, 95% CI: 1.68–5.04). This is supported by a systematic review and meta-analysis done on case-control studies in Iran at different times [5, 30]. It is also supported by pooled analysis of 117 epidemiologic studies [2]. According to one theory, the association between early menarche and BC is caused by breast tissue being exposed to cyclic hormonal stimulation over longer periods [31]. Being exposed for a long time and/or to high levels of estrogen hormone has been linked to an increased risk of BC [32]. Although early menarche is a non-modifiable risk factor for BC, improving early screening, diagnosis, and breast self-examination for those women is possible to reduce the disease's progression. However, only 36.72% of women in Ethiopia Practice breast self-examination as identified by one systematic review and meta-analysis done in 2021 [15]. According to one study, one of the important factors for advanced-stage diagnosis of BC in Ethiopia is a lack of breast self-examination [33]. Therefore, increasing women’s and adolescents’ awareness about BC knowledge and encouraging breast self-examination is important.

### Menopause status

Being in post-menopause was associated with 2.37 times higher odds of developing BC as compared to the pre-menopausal period (AOR = 2.37, 95% CI: 1.67–3.06). This finding is supported by findings of systematic review and meta-analysis done in southeast Asia [34]. However, according to a study done in Iran, there was no association between menopause status and BC [30]. The reason for the discrepancy might be socioeconomic and lifestyle differences. However, the finding of 117 epidemiological analyses of the Lancet report indicates that premenopausal women had a greater risk of BC than postmenopausal women of an identical age [35]. The possibility exists that post-menopause increased risk of BC in this study might be brought on by age-related factors [1, 36]. Therefore, improving post-menopausal women's health-seeking behavior enables early disease detection and timely treatment of BC for better treatment outcomes.

### Family history of cancer

Having a family history of cancer was associated with 2.39 times higher odds of developing BC as compared to their counterpart (AOR = 2.39, 95% CI: 1.29–3.44); which is supported by findings of systematic review and meta-analysis done in southwest Asia and Iran [30, 34]. Therefore, women having a family history of cancer should be closely checked for their health status compared to other communities.

### Body mass index

In the present study, the pooled analysis of odds ratios doesn’t show a significant association between body mass index and odds of developing BC. However, many meta-analyses indicate that there is an association [34]. The reason for the discrepancy might be due to a smaller study in Ethiopia investigating the association between BMI and the odds of BC. High body mass index and BC association is mainly due to obesity-related factors. Obesity raises the risk of BC because of fat tissue that creates an excess of estrogen; which is associated with an increased risk of BC [36, 37]. As a result, it has implications for lifestyle modification.

## Limitations of the study

Even though this study brought recent evidence on determinants of BC in Ethiopian women it has some limitations. Even if many studies indicate oral contraceptive use [5, 25, 38], smoking [39], dietary fiber consumption [40, 41], physical exercise [25, 42], history of abortion [5], drinking alcohol [25], red meat consumption [25, 39], overweight or obesity [25], use of skin lighteners and hair relaxers [43], and a number of parity [34] were found to be associated with BC, the present study didn’t include these variables. This is because of a lack of studies carried out in Ethiopia on the association between the risk of getting BC and those variables. Therefore, further studies should be conducted in Ethiopia by incorporating the above-listed variables.

## Conclusion

The significant determinants of BC in the study area are early menarche, family history of cancer, post-menopausal status, and not ever breastfeeding. Therefore, it is recommended that the Ministry of Health, regional health, zonal and district health departments, and other interested non-profit organizations should work cooperatively to reduce the risk of BC. In addition, the aforementioned factors should be targeted in addressing the problem of BC in Ethiopia by increasing community awareness, promoting breast self-examination, and developing programmes to increase women's knowledge to reduce the increasing burden of BC in Ethiopia.

Furthermore, we recommend that additional studies be conducted in the country to assess the association between BC and oral contraceptive use, obesity, smoking, alcohol consumption, red meat consumption, abortion, physical activity, and dietary fiber consumption, which have not yet been studied in Ethiopia, even if studies from other countries have shown an association.

## List of abbreviations

BMI: body mass index; AOR: adjusted odds ratio; PECOS: population, exposure, comparisons, outcome, study design.

## Conflicts of interest

The authors declare that they have no competing interests.

## Funding

This work was not funded by any organization.

## Author contributions

LKS was involved in conceptualizing, protocol registration process, searching, data extraction, formal analysis, and manuscript writing, and EEC was involved in searching, data extraction, formal analysis, and manuscript writing. All authors reviewed the manuscript.

## Availability of data and materials

The dataset analyzed for this study's findings is available online (https://figshare.com/s/7ec253e86a75d5b4ffd3).

## Figures and Tables

**Table 1.** Research question according to the PECOS framework.

Framework	Description
Population	Ethiopian women
Exposure	Females with specified risk factors
Comparison	Females without specified risk factors
Outcome	Significant determinant of pathologically confirmed BC
Study design	Observational (cross-sectional, case-control, cohort)

**Table 2.** Risk of bias assessment.

Authors	NOS criteria								
	Case definition	Representativeness	Control selection	Definition of controls	Comparability	Ascertainment of exposure	Ascertainment similarity of case and control	Non-response rate	Quality
Tolesa *et al*, 2021 [19]	*	*	0	*	*	*	*	*	8
Hassen *et al*, 2022 [20]	*	*	0	*	*	*	*	*	8
Duche *et al*, 2021 [21]	*	*	0	*	*	*	*	0	7
Letta and Addissie, 2013 [22]	*	*	0	*	*	*	*	*	8
Mengesha and Seme, 2015 [23]	*	*	0	*	*	*	*	*	8
Hassen *et al*, 2021 [24]	*	*	0	*	*	0	0	*	6

**Table 3.** Characteristics of studies included studies.

Author	City	Study period	Setting and control source	The mean age of the case and control respectively	Name of a health institution	Study design	Sample size (case: control ratio)	Determinants	OR	LL	UL
Tolesa *et al*, 2021 [19]	Addis Ababa	2020	Institution based	42.7 (±11.3) 40.7 (±14.6)	Tikur Anbesa Specialized Hospital St. Paul’s Hospital Millennium Medical College	Case-control	248 (1:2)	Age at menarche	4.1	1.84	9.15
								Family history of cancer	2.11	1.04	4.26
Hassen *et al*, 2022 [20]	Addis Ababa	2019	Institution based	42.83 ± 12.06 39.33 ± 11.14	Tikur Anbesa Specialized Hospital	Case-control	460 (1:1)	Age at menarche	3.16	1.78	5.56
								Menopause status	2.34	1.5	3.64
Duche* et al*, 2021 [21]	Addis Ababa	2017	Institution based	43.8 (±12.63) 39.6 (±12.91)	Tikur Anbessa Specialized Hospital St. Paul’s Hospital Millennium Medical College Bethzatha Hospital Korean Hospital	Case-control	220 (1:1)	Menopause status	6.8	1.92	24.16
								Ever breastfeed baby	0.21	0.11	0.42
								Body mass index	5.9	2.16	16.48
Letta and Addissie, 2013 [22]	Addis Ababa	2013	Institution based	39.8 ± 12.5	TIkur Anbessa Specialized Hospital	Case-control	357 (1:2)	Body mass index	4.84	1.82	12.9
Mengesha and Seme, 2015 [23]	Addis Ababa	2015	Institution based	42 ± 9.7 47.7 ± 12.3	TIkur Anbessa Specialized Hospital	Case-control	434 (1:1)	Family history of cancer	2.57	1.5	4.4
								Menopause status	2.4	1.2	4.7
								Ever breastfeed	0.05	0.34	0.87

OR, odds ratio; LL, lower level; UL, upper level

## References

[1] American Cancer Society (2020). Breast Cancer Facts & Figures 2019-2020.

[2] Center of for Disease Control and Prevention (CDC) (2023). What Is Breast Cancer?.

[3] American Cancer Society (2022). Breast Cancer What is Breast Cancer?.

[4] Ngwa W, Addai BW, Adewole I (2022). Cancer in sub-Saharan Africa: a Lancet Oncology Commission. Lancet Oncol.

[5] Khoramdad M, Dodaran MS, Kabir A (2022). Breast cancer risk factors in Iranian women: a systematic review and meta-analysis of matched case–control studies. Eur J Med Res.

[6] International Agency for Research on Cancer (IARC) (2021). Breast cancer awareness month 2021 [Internet]. https://www.iarc.who.int/featured-news/breast-cancer-awareness-month-2021/#:~:text=Breastcancerbecamethemostcommonlydiagnosedcancer,fifthmostcommoncauseofcancerdeathoverall.

[7] IARC (2022). Cancer in sub-Saharan Africa: building local capacity for data production, analysis, and interpretation. Int Agency Res Cancer.

[8] Gbenonsi G, Boucham M, Belrhiti Z (2021). Health system factors that influence diagnostic and treatment intervals in women with breast cancer in sub-Saharan Africa: a systematic review. BMC Public Health.

[9] Morgan GW, Foster K, Huynh V (2018). Improving health and cancer services in low-resource countries to attain the sustainable development goals target 3.4 for noncommunicable diseases. J Glob Oncol.

[10] World Health Organization (WHO) (2014). Cancer – a growing public health concern for Ethiopia [Internet]. https://www.afro.who.int/news/cancer-growing-public-health-concern-ethiopia#:~:text=Cancerisanincreasingpublichealthburdenfor,fourpercentofalldeathsinEthiopia.

[11] World Health Organization (2020). Cancer Ethiopia 2020 Country Profile.

[12] Awedew AF, Asefa Z, Belay WB (2022). National Burden and Trend of Cancer in Ethiopia, 2010 – 2019: a systemic analysis for Global burden of disease study. Sci Rep.

[13] Gebretsadik A, Bogale N, Negera DG (2021). Epidemiological trends of breast cancer in Southern Ethiopia : a seven-year retrospective review. SAGE.

[14] Getachew S, Tesfaw A, Kaba M (2020). Perceived barriers to early diagnosis of breast cancer in south and southwestern Ethiopia : a qualitative study. BMC Womens Health.

[15] Gizachew Y, Id Y, Kassa GM (2021). Breast self-examination practice and its determinants among women in Ethiopia : a systematic review and meta-analysis. PLoS One.

[16] Dagne S, Abate SM, Tigeneh W (2019). Assessment of breast cancer treatment outcome at Tikur Anbessa Specialized Hospital Adult. Eur J Oncol Pharm.

[17] Woldu MA, Legese DA, Abamecha FE (2017). The prevalence of cancer and its associated risk factors among patients cancer science & therapy the prevalence of cancer and its associated risk factors among patients visiting oncology unit, Tikur Anbessa Specialized Hospital, Addis Ababa-Ethiopia. J Cancer Sci Ther.

[18] Palmeri V, Colamesta V, Torre LL (2016). OPE AC evaluation of methodological quality of studies. Senses Sci.

[19] Tolessa L, Sendo EG, Dinegde NG (2021). Risk factors associated with breast cancer among women in Addis Ababa, Ethiopia: unmatched case–control study. Int J Womens Health.

[20] Hassen F, Enquselassie F, Ali A (2022). Association of risk factors and breast cancer among women treated at Tikur Anbessa Specialized Hospital, Addis Ababa, Ethiopia: a case-control study. BMJ Open.

[21] Duche H, Tsegay AT, Tamirat KS (2021). Identifying risk factors of breast cancer among women attending selected hospitals of addis ababa city: Hospital-based unmatched case-control study. Breast Cancer Targets Ther.

[22] Letta G, Addissie A (2013). Magnitude of breast and cervical cancer and associated risk factors of breast cancer in Addis Ababa, Ethiopia, Masters Thesis, Addis Ababa University, Addis Ababa. Addis Abeba Univ Res Repos.

[23] Mengesha H, Seme A (2015). Association of non breast feeding and breast cancer among patients on chemotherapy and radiotherapy at Tikur Anbessa Specialized Hospital : a case-control study, Masters Thesis, Addis Abeba University, Addis Ababa. Addis Abeba Univ Res Repos.

[24] Hassen F, Enquselassie F, Ali A (2021). Socio-demographic and haematological determinants of breast cancer in a Tertiary Health Care and Teaching Hospital in Addis Ababa, Ethiopia. Ethiop J Heal Dev.

[25] Poorolajal J, Heidarimoghis F, Karami M (2021). Factors for the primary prevention of breast cancer: a meta-analysis of prospective cohort studies. J Res Health Sci.

[26] Bernier MO, Bossard N, Ayzac L (2000). Breast feeding and risk of breast cancer: a meta-analysis of published studies. Eur Soc Hum Reprod Embrol.

[27] Stordal B (2023). Breastfeeding reduces the risk of breast cancer: a call for action in high-income countries with low rates of breastfeeding. Cancer Med.

[28] Kebebe T, Assaye H (2017). Intention, magnitude and factors associated with bottle feeding among mothers of 0–23 months old children in Holeta town, Central Ethiopia: a cross sectional study. BMC Nutr.

[29] Kebede EM, Seifu B (2021). Breastfeeding and employed mothers in Ethiopia: legal protection, arrangement, and support. Int Breastfeed J.

[30] Shamshirian A, Heydari K, Shams Z (2020). Breast cancer risk factors in Iran: a systematic review & meta-analysis. Horm Mol Biol Clin Investig.

[31] Olsson HL, Olsson ML (2020). The menstrual cycle and risk of breast cancer: a review. Front Oncol.

[32] National Cancer Institute (2015). Cancer causes and prevention [Internet]. https://www.cancer.gov/about-cancer/causes-prevention/risk/hormones#:~:text=Studieshavealsoshownthatawoman’srisk,linkedtoanincreasedriskofbreastcancer.

[33] Tesfaw A, Tiruneh M, Tamire T (2021). Factors associated with advanced-stage diagnosis of breast cancer in north-west Ethiopia: a cross-sectional study. Ecancermedicalscience.

[34] Nindrea RD, Aryandono T, Lazuardi L (2017). Breast cancer risk from modifiable and non-modifiable risk factors among women in Southeast Asia: a meta-analysis. Asian Pac J Cancer Prev.

[35] Hamajima N, Hirose K, Tajima K (2012). Menarche, menopause, and breast cancer risk: individual participant meta-analysis, including 118 964 women with breast cancer from 117 epidemiological studies. Lancet Oncol.

[36] Łukasiewicz S, Czeczelewski M, Forma A (2021). Breast cancer – epidemiology, risk factors, classification, prognostic markers, and current treatment strategies – an updated review. MDPI Cancers.

[37] Liu XZ, Pedersen L, Halberg N (2021). Cellular mechanisms linking cancers to obesity. Cell Stress.

[38] Rushton L, Jones DR (1992). Oral contraceptive use and breast cancer risk: a meta‐analysis of variations with age at diagnosis, parity and total duration of oral contraceptive use. BJOG Int J Obstet Gynaecol.

[39] Namiranian N, Moradi-Lakeh M, Razavi-Ratki SK (2014). Risk factors of breast cancer in the eastern mediterranean region: a systematic review and meta-analysis. Asian Pacific J Cancer Prev.

[40] Farvid MS, Spence ND, Holmes MD (2020). Fiber consumption and breast cancer incidence: a systematic review and meta-analysis of prospective studies. Cancer.

[41] Chen S, Chen Y, Ma S (2016). Dietary fibre intake and risk of breast cancer: a systematic review and meta-analysis of epidemiological studies. Oncotarget.

[42] Pizot C, Boniol M, Mullie P (2016). Physical activity, hormone replacement therapy and breast cancer risk: a meta-analysis of prospective studies. Eur J Cancer.

[43] Brinton LA, Figueroa JD, Ansong D (2018). Skin lighteners and hair relaxers as risk factors for breast cancer: results from the Ghana breast health study. Carcinogenesis.

